# Epidemiological connectivity between humans and animals across an urban landscape

**DOI:** 10.1073/pnas.2218860120

**Published:** 2023-07-14

**Authors:** James M. Hassell, Dishon M. Muloi, Kimberly L. VanderWaal, Melissa J. Ward, Judy Bettridge, Nduhiu Gitahi, Tom Ouko, Titus Imboma, James Akoko, Maurice Karani, Patrick Muinde, Yukiko Nakamura, Lorren Alumasa, Erin Furmaga, Titus Kaitho, Fredrick Amanya, Allan Ogendo, Francesco Fava, Bryan A. Wee, Hang Phan, John Kiiru, Erastus Kang’ethe, Sam Kariuki, Timothy Robinson, Michael Begon, Mark E. J. Woolhouse, Eric M. Fèvre

**Affiliations:** ^a^Global Health Program, Smithsonian’s National Zoo and Conservation Biology Institute, Washington, DC 20008; ^b^Department of Epidemiology of Microbial Diseases, Yale School of Public Health, CT 06510; ^c^Institute of Infection, Veterinary and Ecological Sciences, University of Liverpool, Liverpool L69 3BX, United Kingdom; ^d^Usher Institute, University of Edinburgh, Edinburgh EH16 4SS, United Kingdom; ^e^International Livestock Research Institute, 00100 Nairobi, Kenya; ^f^Centre for Immunity, Infection and Evolution, University of Edinburgh, Edinburgh EH9 3FL, United Kingdom; ^g^Department of Veterinary Population Medicine, College of Veterinary Medicine, University of Minnesota, St. Paul, MN 55108; ^h^Nuffield Department of Clinical Medicine, University of Oxford, Oxford OX3 7BN, United Kingdom; ^i^Faculty of Medicine, University of Southampton, Southamton SO17 1BJ, United Kingdom; ^j^University of Nairobi, 00625 Nairobi, Kenya; ^k^Kenya Medical Research Institute, 00200 Nairobi, Kenya; ^l^National Museums of Kenya, 00100 Nairobi, Kenya; ^m^Faculty of Veterinary Medicine, Hokkaido University, Sapporo 060-0818, Japan; ^n^Department of Epidemiology, Columbia University, New York, NY 10032; ^o^Veterinary Services Department, Kenya Wildlife Service, 00100 Nairobi, Kenya; ^p^Department of Environmental Science and Policy, Università degli Studi di Milano, 20133 Milan, Italy; ^q^Food and Agriculture Organization of the United Nations, 00153 Rome, Italy

**Keywords:** disease ecology, urbanization, pathogen spillover, interface, one health

## Abstract

Urbanization is predicted to be a key driver of disease emergence through human exposure to novel, animal-borne pathogens. However, while we suspect that urban landscapes are primed to expose people to novel animal-borne diseases, evidence for the mechanisms by which this occurs is lacking. To address this, we studied how bacterial genes are shared between wild animals, livestock, and humans (n = 1,428) across Nairobi, Kenya—one of the world’s most rapidly developing cities. Applying a multilayer network framework, we show that low biodiversity (of both natural habitat and vertebrate wildlife communities), coupled with livestock management practices and more densely populated urban environments, promotes sharing of *Escherichia coli*–borne bacterial mobile genetic elements between animals and humans. These results provide empirical support for hypotheses linking resource provision, the biological simplification of urban landscapes, and human and livestock demography to urban dynamics of cross-species pathogen transmission at a landscape scale. Urban areas where high densities of people and livestock live in close association with synanthropes (species such as rodents that are more competent reservoirs for zoonotic pathogens) should be prioritized for disease surveillance and control.

## Significance

In this study, we conducted a comparative analysis of bacterial gene sharing across urban human and animal populations. Because the genes we targeted (called “mobile genetic elements”) are shared horizontally between bacteria, their population structure can be used to infer bacterial transmission between hosts. By uncovering characteristics of human and animal populations and the urban environments in which they live that promote cross-species sharing of *Escherichia coli*–borne genes, this study sheds important light on the conditions that could facilitate spillover of pathogens. A clear understanding of the socioecological determinants of cross-species transmission is crucial for design of public health interventions aimed at limiting human exposure to novel animal-borne pathogens in rapidly developing cities.

Most human emerging infectious diseases (EIDs) originate in animals (termed zoonoses), and the rate at which novel infections spillover from wildlife and livestock into people is predicted to increase (1). Rapid, unplanned urbanization is characteristic of cities in lower and middle-income countries (2). In the tropics, cities are a crucible for activity and interaction between animals and humans: Wealthy live alongside poor; livestock live alongside people and a rich assortment of wildlife, where waste is often poorly disposed of; and animal products are traded along complex supply chains. Dynamic interfaces between wild animals, domestic animals, and humans provide a route by which people living in major cities can be exposed to animal-borne pathogens in an environment where pathogenic microorganisms can quickly amplify and spread along human networks of connectivity (3). The potential for these events to cause large-scale global disruption is evident from the SARS-CoV-2 pandemic. The emergence of novel human pathogens in major urban centers (e.g., severe acute respiratory syndrome coronavirus, severe acute respiratory syndrome coronavirus 2, highly pathogenic avian influenza H5N1, and novel bacterial strains demonstrating antimicrobial resistance) highlights the importance of urbanization as a driver of disease emergence (4–7). Efforts to study the circumstances under which these diseases emerged in people have provided an understanding of behavioral risk, particularly as it relates to interaction with urban trade in animals and their products (8–10). Yet, little is known of how environmental and socioeconomic processes, instigated by urban development, influence the transmission and emergence of pathogens between urban populations of wildlife, livestock, and people. A better understanding of cross-species transmission at animal–human interfaces in urban environments is urgently required to determine factors that generate entry points for zoonotic pathogens into humans and to inform targeted surveillance efforts for early detection and response to EIDs in people and animals (11).

Historically, studies aimed at understanding the socioecological drivers of zoonotic pathogen emergence have typically looked at such issues from a continental/global scale, with limited explanatory potential. Empirical work, nested within landscapes, is required to study the small-scale processes that underly spillover and inform targeted risk and control measures. By capturing the structural complexity and heterogeneous mixing of individuals within a population, epidemiological networks can be used to investigate factors affecting transmission, while also providing a realistic framework for modeling pathogen spread between hosts (12–14). Network analysis is gaining traction as a method with which to investigate transmission dynamics within complex multihost systems, through quantitative measures of pathogen transmission derived from the population structure of microorganisms (15–18). Patterns of pathogen sharing between pairs of individuals of the same or different host species are used to quantify the likelihood of transmission between hosts, which can be interpreted as the potential of a host to spread pathogens relative to other nodes in the network (16, 19). Networks formulated in this way [referred to as transmission potential networks (TPNs)] provide an elegant method by which to infer the role and relative importance of different hosts in pathogen transmission and identify points at which control efforts could be targeted (16).

*Escherichia coli* is commonly used as a model organism to infer the dynamics of pathogen transmission between hosts. However, relatively slow evolutionary rates and clonal structure complicate the phylogenetic interpretation of *E. coli* transmission at fine spatial and temporal scales (20). In recent work, the distribution of genes borne on mobile genetic elements (MGEs) within prokaryote genomes has been used to infer bacterial transmission and epidemiological connectivity between hosts, independently of their taxonomic distance (21, 22). Building on this, we constructed TPNs from pairwise sharing of MGEs belonging to commensal *E. coli* to study the potential for transmission to occur between humans and the animals with which they coexist across the city of Nairobi, Kenya. This rapidly developing urban landscape is representative of many other cities in Africa, making it an ideal real-world system in which to explore how urban development and people’s interactions and coexistence with animals influences pathogen transmission across species.

**Fig. 1.**
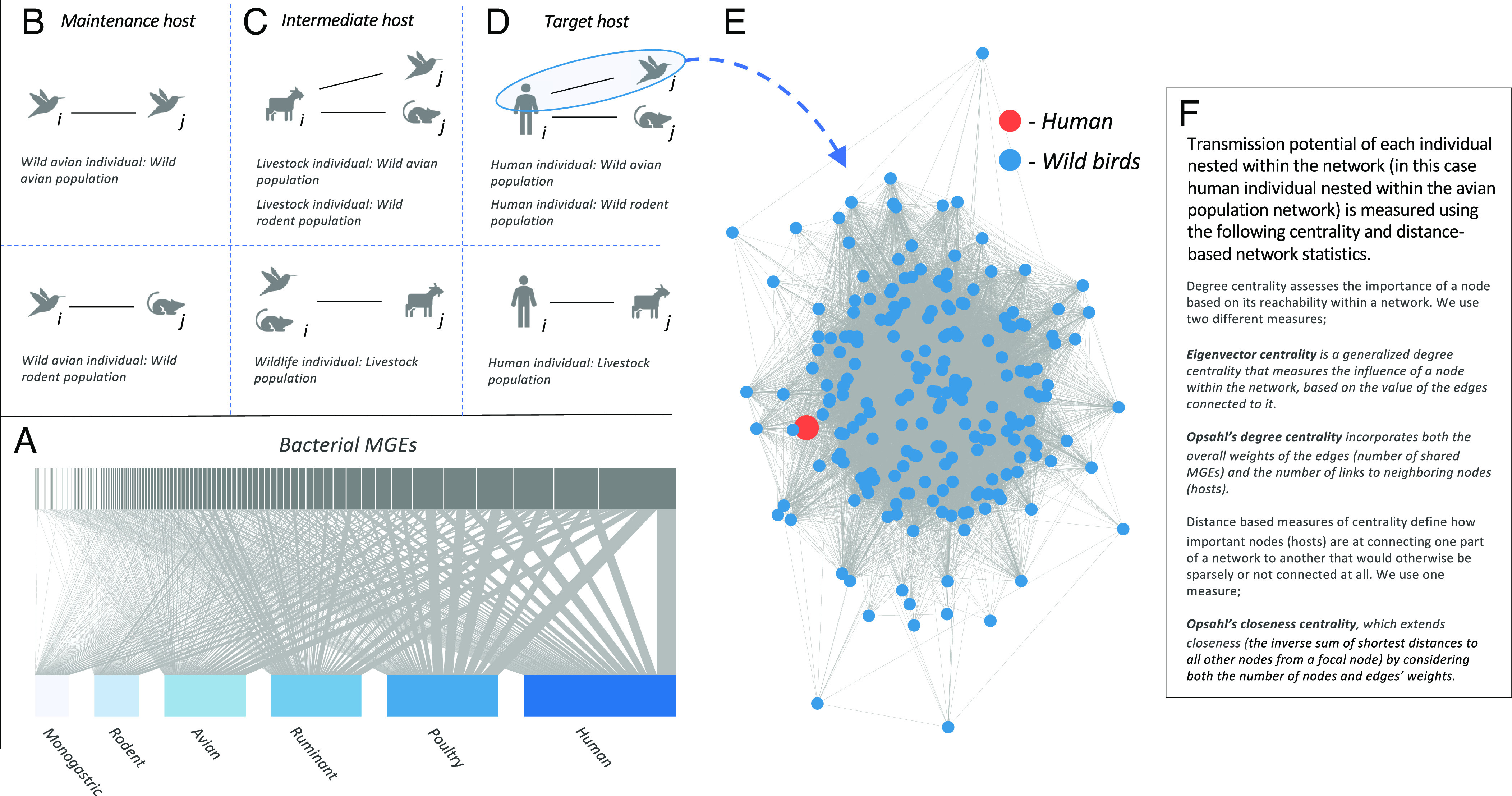
Approach to developing multilayer TPNs that link wildlife, livestock, and human populations across Nairobi by sharing of *E. coli*–borne MGEs. (*A*) Bipartite network linking animal and human host compartments with their MGE communities. (*B–D*) Pairwise comparisons of shared MGEs, grouped by the putative epidemiological role of each host group within a multispecies framework for urban disease emergence. For each member of taxon*_i_*, a matrix of shared MGEs was created with all individuals of taxon*_j_* and used to generate a bipartite network. Each bipartite network was projected to a weighted unipartite network (multilayer transmission potential network—mTPN). (*E*) This effectively nested each member of taxon*_i_* within an mTPN including all members of taxon*_j_* and enabled us to compare the relative importance of individual hosts in sharing bacterial genes across taxonomic interfaces. (*F*) Centrality and distance-based network statistics that best captured pairwise sharing of MGEs between each individual and all other wildlife hosts within each mTPN, were then used to represent transmission potential for each individual host.

Our approach was to first create a highly structured and informative sample frame that captures wildlife–livestock–human interfaces across the city (20). Integrating network analysis with statistical modeling, we then asked how well a suite of biotic and human variables that are hypothesized to influence host dynamics and contact rates explain transmission potential between wildlife, livestock, and humans across the city. Transmission potential—a term we use to describe how connected hosts are to one another through sharing of *E. coli*–borne MGEs—is measured using centrality statistics that capture pairwise sharing of MGEs between individuals within TPNs (see Fig. 1*F* and *SI Appendix*, Fig. S1 for further details). The concept of transmission potential is increasingly used in disease ecology where network analysis is applied to investigate infectious disease transmission, although we apply a more exact definition in this study (16, 19, 23) We begin by assessing whether MGE sharing in our dataset is epidemiologically relevant by testing hypotheses of isolation by distance and isolation by environment against a null model of unstructured sharing of MGEs between hosts. Our next set of hypotheses—which follow—was framed within and across taxonomic interfaces, through the lens of existing epidemiological frameworks for disease emergence (24–26) in which wildlife form a maintenance community of microorganisms from which novel zoonotic pathogens can spillover to intermediate hosts (such as livestock) or humans. Spatial overlap and density of reservoir and intermediate hosts, which determine interspecies contact rates, cross-species transmission, and amplification of many directly transmitted pathogens, are typically related to patterns of development and socioeconomics in urban settings. Since provision of resources (e.g., availability of natural or artificial habitat for foraging or reproduction) is a key driver of interspecies wildlife contact, we expected TPNs to show that pairwise sharing of MGEs is higher between wildlife taxa inhabiting less biodiverse habitats with more restricted ecological niches, and in the presence of agricultural resources that would be expected to promote contact between wildlife. Since livestock are one such agricultural resource that attracts wildlife, we also expected wildlife and livestock to share more MGEs in settings characterized by lower biosecurity urban livestock-keeping practices. For example, manure has been identified as a potential interface for bacterial transmission between wild birds and rodents. Finally, we expected pairwise sharing of MGEs to be highest between animals and humans in areas of the city where population growth and density of people and their livestock is concentrated, promoting increased contact and transfer of bacterial genes.

## Results

Fecal samples (n = 2,081) were collected from humans (n = 333), 13 livestock species (n = 677), 63 wildlife species (birds and rodents, n = 695), and their shared environment (n = 277) in 99 households across Nairobi (20). Households, participating in the UrbanZoo project, were selected to capture variation in human sociodemographics, livestock-keeping practices, urban land use, and wildlife assemblages across the city (*SI Appendix*, Fig. S1). Following previous work, we chose household compounds—people’s houses and private land—as representative habitat patches for variation in urban environments and important human–animal interfaces (27–30). A total of 1,487 *E. coli* isolates, each representing a different host individual, underwent whole-genome sequencing (WGS) and were subsequently filtered for non–*E. coli* and isolates with a genome size larger than 6 megabases. A total of 1,428 *E. coli* sequences with genes carried on MGEs in the accessory genome were considered in further analyses.

### Vertical Transmission and Socioecological Factors Influence Population Structure of *E. coli*–Borne MGEs.

Our approach assumes that the mechanisms underlying sharing of *E. coli*–borne MGEs between hosts are reflective of the potential for these individuals to be part of the same transmission chain. That is, we expected hosts with similar MGE profiles to have acquired bacterial genes through direct transmission or to share ecological or physiological characteristics that promote acquisition of bacteria with similar MGEs (e.g., characteristics such as foraging traits that would lead individuals to be infected at a common source). To better characterize whether pairwise sharing of MGEs is epidemiologically meaningful, we began by assessing whether MGE sharing between hosts represents transmission of *E. coli* through co-occurrence of hosts in space and time (isolation by distance, in which case hosts that are well mixed in a given area would be expected to have high MGE sharing) or being part of the same broad transmission chains (isolation by environment, for example, exposure of hosts to a common source or broader coevolutionary processes between the *E. coli* core genome and their hosts as opposed to direct transmission of MGEs).

A distance-based variant of canonical variation partitioning analysis (dbRDA) was used to test for associations between variation in MGE assemblages (represented as a matrix of pairwise Jaccard distances) with micro- and macro-scale factors that would be expected to determine the distribution of *E. coli*–borne MGEs in hosts across Nairobi, against a null model of unstructured MGE sharing between hosts (e.g., MGE sharing was random with respect to the considered micro- and macro-scale factors). Four groups of explanatory variables were included, representing i) the geographical distance between each host at the time of sampling; ii) bacterial population structure represented by the multilocus sequence type (MLST) of the core genome of each *E. coli* isolate (as opposed to MGEs); iii) the host taxa from which each bacterial isolate was cultured, and iv) the ecological and anthropogenic characteristics of each household (wildlife diversity, livestock-keeping practices, affluence, and human density).

**Fig. 2.**
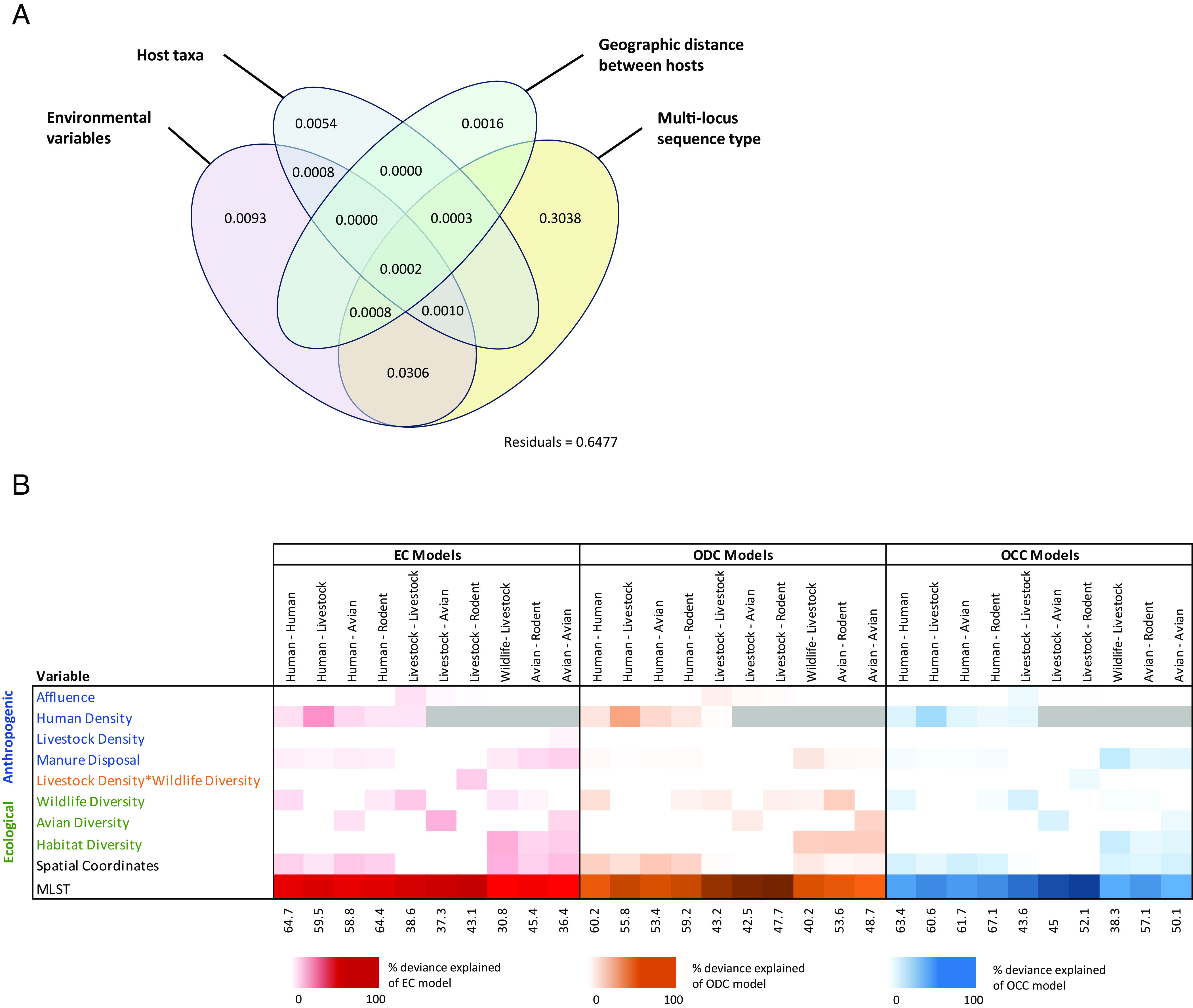
Variables predicting population structure and cross-species sharing of bacterial MGEs at taxonomic animal-animal and animal-human interfaces across Nairobi. (*A*) Results of variance partitioning, showing the variance in MGE community structure explained by variables representing evolutionary, host ecology, and environmental factors. These values were derived from a distance-based redundancy analysis (dbRDA) performed on a distance matrix of MGE sharing between hosts. (*B*) % deviance explained by environmental variables included within generalized additive models (GAMs) exploring the impact of biotic and anthropological environmental variables that are hypothesized to influence host dynamics and contact rates on centrality within multilayer TPNs (mTPNs). The results from three different categories of models are presented, each representing a different measure of centrality across ten pairwise taxonomic networks. In EC, ODC, and OCC models, eigenvector centrality, Opsahl degree centrality, and Opsahl closeness centrality were measured as the response variable, respectively.

Variance partitioning within the dbRDA revealed that the structure of MGE assemblages was best explained by a combination of bacterial population structure, host/environmental factors, and geographic distance between hosts (R^2^ adj = 0.36) (Fig. 2*A*). Unsurprisingly, MLST accounted for most of the total variance in the dataset (30.6%), suggesting that organismal spread (i.e., that the spread of the whole organism is more important in shaping the spread of MGEs than transduction/transformation) plays a considerable role in MGE sharing between bacteria in different hosts. Host taxa and environmental factors collectively accounted for 4.9% of variance (0.7% and 4.3%, respectively), while the spatial distribution of hosts accounted for minimal variance in MGE assemblages (0.2%). These results indicate that MGE sharing is epidemiologically meaningful and probably reflective of hosts being part of the same broad *E. coli* transmission chains.

### Bacterial Gene Sharing Varies between Wildlife, Livestock, and Humans.

**Fig. 3.**
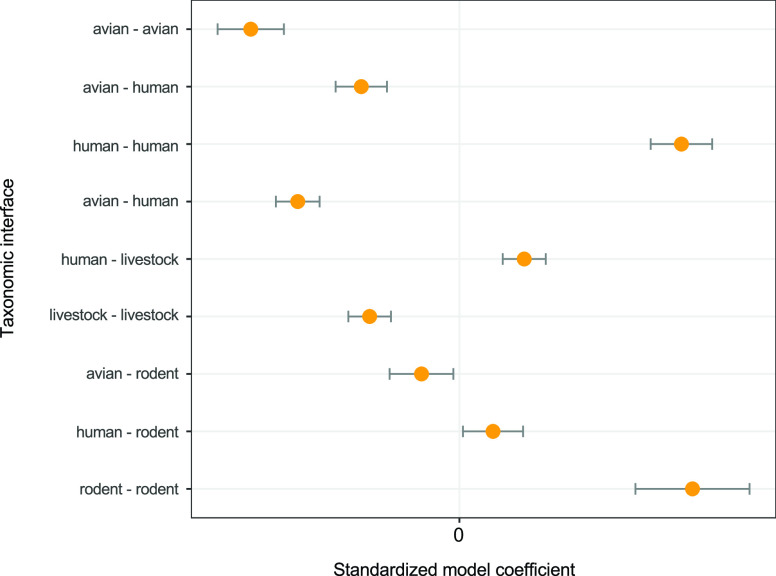
Estimates from an exponential random graph model, comparing the likelihood of hosts sharing more than one MGE within and between taxonomic groups within a unipartite network representing all hosts across the city. The livestock–rodent taxonomic interface is set as a reference level within the model, with which to compare other interfaces.

In assessing epidemiological connectivity between urban populations of animals and humans, we began by comparing bacterial gene sharing between host taxa (wildlife, livestock, and humans) across the city. To do this, a bipartite network linking all hosts and their bacterial MGEs was projected to a weighted unipartite network in which each node represented an individual connected with all other hosts in the network by their number of shared MGEs. An exponential random graph model (ergm) was then used to examine the extent to which the taxonomic group influenced the likelihood of MGE sharing between hosts within the network, as opposed to a null model where MGE sharing is only determined by the density of the network (e.g., the probability of a link existing between any two individuals in the network, without respect to the taxonomic group). At a city-wide scale, we found that sharing of MGEs between rodents and between humans was consistently more likely to occur when compared to other taxa, and rodents, humans, and livestock were significantly more likely to share MGEs with one another than birds (Fig. 3).

### Low Diversity of Natural Habitat Predicts Transmission Potential between Wildlife (Putative Reservoir Hosts).

To explore epidemiological connectivity between urban populations of animals and humans in more depth, we examined factors affecting how connected people, livestock, and wildlife are within multilayer TPNs (mTPNs) composed of pairwise sharing of bacterial genes (see *SI Appendix*, Fig. S1 for an explanation of our conceptual model for zoonotic spillover in urban settings and variables used to explore this and Fig. 1 for how mTPNs were structured). Briefly, this approach was used to construct networks that represented how each sampled individual was embedded in networks composed of a specific taxonomic group (e.g., a particular wildlife sample within wildlife networks or a wildlife sample within livestock networks). Within this framework, we tested our first hypothesis for how the effects of host population structure and resource provision increase transmission potential within Nairobi’s wildlife population.

We began by building bipartite networks linking each individual wild avian host with all other wildlife hosts in each of the two groups—wild birds and rodents—by their shared MGEs (Fig. 1*A*). In brief, a separate bipartite network was constructed for each bird and all other birds or rodents across the city, resulting in 2(*n*) networks (where *n* = 243, the total number of birds in the dataset). Bipartite networks were projected to weighted unipartite networks (Fig. 1 *B* and *E*), whereby nodes represent individual samples, and connections between nodes represent the number of shared MGEs. We then identified summary network statistics that best captured pairwise sharing of MGEs between each individual and all other wildlife hosts within each mTPN. Following Wardeh et al. (31), 13 different epidemiologically relevant network statistics representing the centrality of each bird within the larger avian or rodent network were calculated (*SI Appendix*, Table S1). Eigenvector centrality (EC) was selected for further analyses because it has been used to quantify transmission potential in epidemiological studies and has been shown to correlate closely with causal inference (16, 32). Additional measures of centrality best suiting our dataset were chosen using Principal Component Analysis (PCA) by selecting measures that explained the highest variation in the first principal component when averaged across all networks. Opsahl degree centrality (ODC) and Opsahl closeness centrality (OCC) (mean contributions to first principal component = 10.97% and 10.57%, respectively) emerged as the two measures that best described variation across all networks. ODC accounts for the number and strength of direct connections made by each individual in the network, and OCC accounts for many steps away an individual is from all others across the network. In our dataset, individuals with high EC and ODC can be considered members of “super-sharing” clusters for MGEs—belonging to groups of hosts within that network that are more likely to share the same genes, and are therefore at closer transmission distance to one another whether directly or through exposure to a common source. Those with high OCC tend to share genes with others across the entire network and may therefore be at closer transmission distance to the network as a whole (33). Collectively, high values of our selected centrality measures (EC, ODC, and OCC) therefore indicate that an individual has high transmission potential within the network in which it is positioned. Fig. 1*F* and *SI Appendix*, Fig. S1 visually conceptualize transmission potential for the purposes of our study.

**Fig. 4.**
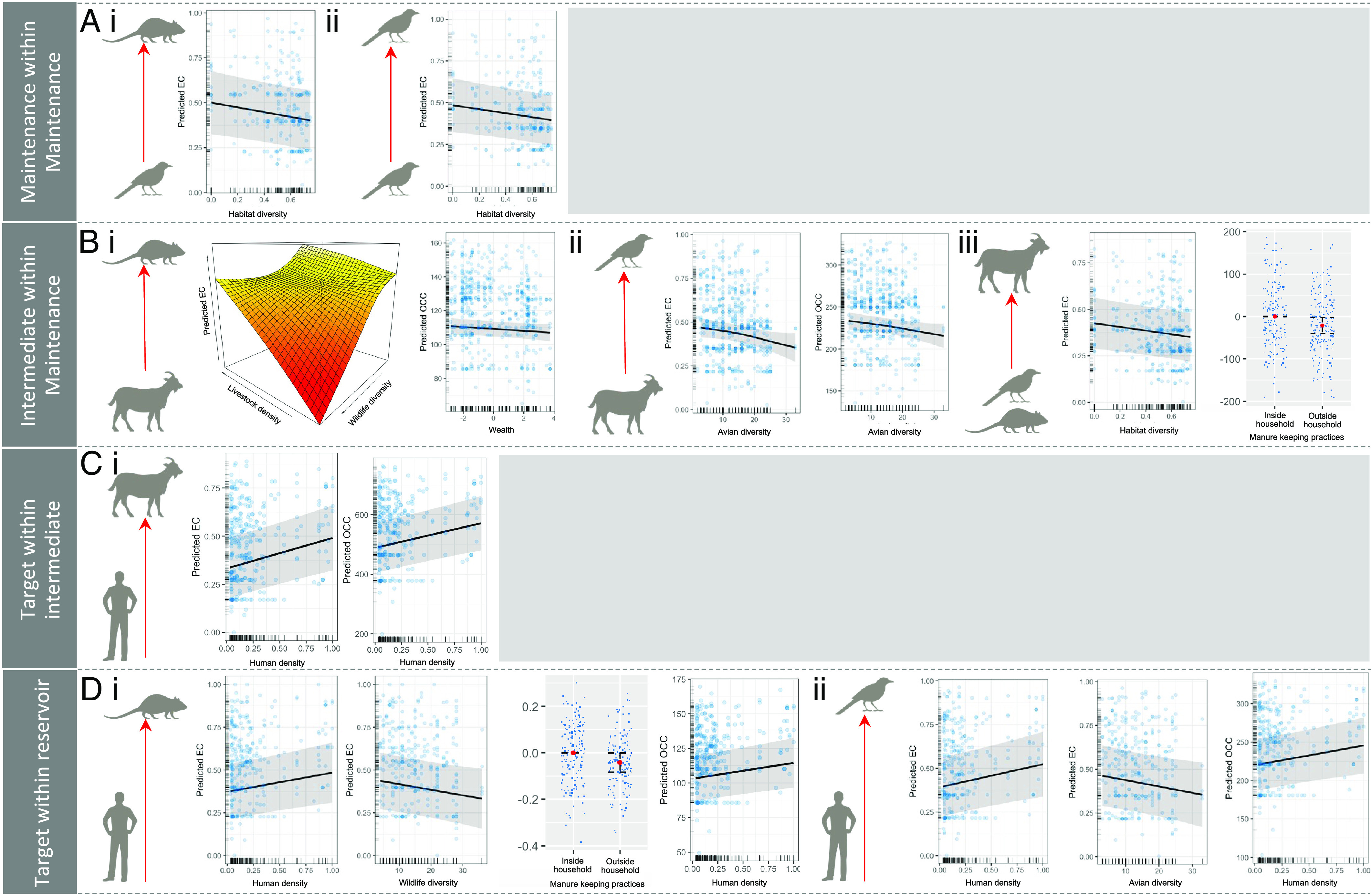
Statistically significant predictors of degree and closeness centrality within mTPNs across epidemiologically relevant taxonomic interfaces in Nairobi. (*A*) Factors influencing centrality of maintenance within maintenance hosts: i) centrality of individual birds with the city’s rodent network and ii) individual birds within the city’s avian network. (*B*) Factors influencing centrality of intermediate within maintenance hosts: i) centrality of individual livestock within the city’s rodent network, ii) individual livestock within the city’s avian network, and iii) individual birds and rodents within the city’s livestock network. (*C*) Factors influencing centrality of target within intermediate hosts: i) centrality of individual people within the city’s livestock network. (*D*) Factors influencing centrality of target within maintenance hosts: i) centrality of individual people within the city’s rodent network and ii) individual people within the city’s avian network.

Measures of avian centrality calculated for the focal individual within each avian and rodent bacterial gene network were then regressed against vertebrate diversity at the sample location (avian diversity for models in which avian centrality was the response and overall wildlife diversity for models in which rodent centrality was the response) and proxies for the availability of urban resources—such as breeding and foraging habitat—within household compounds using generalized additive models (GAMs), while controlling for geographical distance between hosts (and therefore spatial autocorrelation) and bacterial population structure (MLST included as a random effect) (see Fig. 1*B* for a summary of the deviance explained by all models). Each of these models was compared against a null model in which avian centrality within the avian and rodent networks was regressed against only the random effects of spatial autocorrelation and bacterial population structure. Centrality of wild birds within the avian and rodent networks was best explained by natural habitat diversity (represented by Simpson’s diversity index of quantified variation of biological habitat within households), avian or wildlife diversity, livestock density (EC models only), management of livestock waste (EC and OCC models), and geographic distance between hosts and MLST (total deviance explained by avian models: EC = 36.4%, ODC = 48.7%, and OCC = 50.1%; rodent models: EC = 45.4%, ODC = 53.6%, and OCC = 57.1%). With the exception of MLST which described most of the deviance within each model, habitat diversity was the only statistically significant predictor of centrality in all models; as natural habitats within households became less diverse, the transmission potential of wild birds within rodent and avian networks increased (Fig. 4*A*).

### Low Diversity of Natural Habitat and Vertebrate Biodiversity, Socioeconomics, and Management of Livestock Waste Predict Transmission Potential between Wildlife and Livestock (Putative Reservoir and Intermediate Hosts).

Next, we investigated transmission potential between the city’s wildlife and livestock populations. A set of unipartite mTPN networks was generated for each individual domestic animal within the avian network and rodent network 2(*n*) (n = 561, the total number of livestock in the dataset) and a single set of networks for each wild animal (avian and rodent) within the livestock network 1(*n*) (n = 369, the combined number of birds and rodents in the dataset). Because we expected variation in host assemblages and resource provision associated with livestock-keeping practices (such as livestock feed and manure management) to generate entry points for bacterial genes to cross between wildlife and domestic animals, EC, ODC, and OCC values were calculated and regressed against the same set of proxies. Centrality of livestock within the city’s avian network was best described by the wealth status of each house household, avian diversity, and MLST. After MLST, avian diversity explained the most deviance and had significant negative effects on all centrality measures (EC: 3.11%, *P* < 0.01; ODC: 0.96%, *P* < 0.05; OCC: 1.8%, *P* < 0.01). When measured as ODC, wealth was also a weakly significant predictor of centrality. Transmission potential of bacterial MGEs between livestock and wild birds was therefore highest when livestock coexisted with less diverse avian assemblages, with some evidence to suggest that transmission potential was also highest in lower-income areas of the city (Fig. 4*C*).

Livestock centrality within the city’s rodent network was best predicted by an interaction term between livestock density and wildlife diversity, wealth and MLST (EC and OCC models), and wildlife diversity, wealth, and MLST (ODC model). The interaction between livestock density and wildlife diversity had a weakly significant effect (*P* < 0.05) on centrality (EC model only, deviance explained = 2.05%), as did wildlife diversity (ODC model only) and wealth (OCC model only). Although not equally supported across measures of centrality, these results suggest that transmission potential between livestock and rodents was higher in lower-income areas of the city with high densities of livestock, and this effect was to some extent magnified in the presence of low diversity wildlife assemblages (Fig. 4*D*).

Centrality of individual birds and rodents within the livestock network was best described by wildlife diversity, habitat diversity, manure practices, and geographical distance between hosts and MLST. Wildlife inhabiting less biologically diverse habitats were significantly more central within the city’s livestock network (deviance explained: EC = 3.27%; ODC = 2.23%; OCC = 2.47%; *P* < 0.01 across all models), as were wildlife existing in the presence of livestock waste (EC and OCC models) (Fig. 4*E*).

### Vertebrate Biodiversity and Human Density Predict Transmission Potential between Humans and Animals (Putative Target and Intermediate/Reservoir Hosts).

Finally, we applied our mTPN approach to test the hypothesis that variation in livestock-keeping practices, household sociodemographic characteristics, and the assemblages of wildlife with which people exist generate entry points for bacteria and their genes to cross between animals and humans.

3(*n*) (n = 293, the total number of people represented in the dataset) unipartite networks were generated, linking individual human hosts with all animal hosts belonging to each of three taxonomic compartments (livestock, wild birds, and rodents). The best-fit models for human centrality within the city’s livestock mTPN included the human density of each household, manure management practices, geographical distance between individuals, and MLST. After MLST, human density was the second most important predictor in all models and had a strongly significant positive effect on all measures of human centrality, indicating that people living at higher densities received and transmitted more bacterial genes with the city’s livestock population (deviance explained: 4.3% (EC), 3.94% (ODC), 3.89% (OCC); *P* < 0.001) (Fig. 3*F*). Human centrality within the city’s avian and rodent mTPNs was best described by human density, avian or wildlife diversity, management of livestock waste, geographical distance between hosts, and MLST. Human density had a significant (*P* < 0.01) positive effect on all measures of human centrality within avian and rodent mTPNs, while avian diversity had a significant negative effect on human centrality within the EC and ODC avian models. As such, people living at higher densities and coexisting with less diverse avian assemblages were more central within the avian network (Fig. 4 *G* and *H*). When measured using EC, management of livestock waste was also a significant predictor for human centrality within avian and rodent mTPNs—in these models, human transmission potential with wildlife was highest in areas of the city where people lived at high densities and in environments with low diversity wildlife communities where livestock manure was present in the environment.

Since MGEs appear at different frequencies in our dataset, it is likely that certain “cosmopolitan” MGEs that are widely distributed across host taxa contribute disproportionately to gene sharing, meaning that hosts carrying these genetic elements are likely to have a higher centrality within each multilayer mTPN. To account for this, and check that the relationships described above were not biased by urban conditions that favor cosmopolitan MGEs, we reformulated each multilayer mTPN and GAM using a reduced dataset in which MGEs that appeared in similar proportions across all taxonomic groups were removed. Our results (which are presented in *SI Appendix*, Table S4) remain broadly unchanged, providing confidence that the relationships described above represent factors associated with transmission potential between wildlife, livestock, and humans in Nairobi.

## Discussion

For animal-to-human spillover and subsequent spread of a pathogen to occur, a complex set of epidemiological, ecological, and behavioral conditions that influence the composition, infection dynamics, and contact rates within and between host populations must align (26). These conditions could be met through the dramatic socioecological changes that occur because of rapid, unplanned urban development—such as habitat modification, close contact with domestic animals, and social disparity (and resulting health inequalities). By applying a network-based approach for measuring bacterial MGE sharing across taxonomic interfaces to an extensive and uniquely comprehensive dataset collected across one of the world’s most rapidly developing cities, our analyses identified characteristics of human and animal populations and the urban environments in which they live that increase exchange of mobile bacterial genes. These findings provide empirical support for hypotheses linking resource provision and the spatial distribution of hosts to urban dynamics of bacterial gene transfer at a landscape scale. Even though spillover risk is complex, dynamic, and pathogen specific, such empirical data can help us understand facets of the disease emergence processes that could make zoonotic spillover more likely to occur (such as human–animal contact) from a scale at which humans operate.

### Urban Transmission Dynamics.

Provision of supplemental resources has a considerable impact on the community composition and spread of infectious diseases within wildlife populations. Variation in resource provision occurs widely across urban environments, where changes in urban land use and agricultural practices (such as livestock keeping) dictate the structure of wildlife assemblages, leading to aggregations of urban-adapted wildlife species and their parasites (34). In finding that low diversity of urban habitat is associated with increased transmission potential within and between wildlife taxa across Nairobi (avian–avian and avian–rodent interfaces), our results correspond with a considerable body of literature demonstrating the impacts of urban land use on transmission dynamics within wildlife populations (35–38). Increased competition for resources in less biologically diverse parts of Nairobi where natural food sources are restricted presumably influences the density and rates of intra- and interspecific contact between wild animals, which could result in individuals sharing more bacterial genes with wildlife belonging to the same or different taxa. For density-dependent pathogens, amplification through enhanced transmission is expected to occur when density and contact rates between hosts increase, as is the case for hantavirus and Lassa virus infection in urban rodents (39, 40), and Hendra virus among aggregations of fruit bats in Australian cities (41). Because less biologically diverse urban habitats also support higher abundances of taxa such as *Rattus* and *Mus* Spp. that carry more known zoonotic pathogens [a characteristic of Nairobi and urban environments worldwide (27, 34, 42), loss of natural habitat through urban development would be expected to promote higher prevalence of directly transmitted zoonotic pathogens within wildlife reservoirs.

We also provide insight into the urban conditions under which transmission potential between wildlife and livestock (reservoir and intermediate hosts) is highest. In rapidly developing cities in Africa where small-scale livestock keeping is practiced ubiquitously, domestic animals and their waste represent a rich resource for synanthropic species. Comparatively low biosecurity in these environments also means that contact between livestock, livestock products (e.g., waste), and wildlife utilizing these resources for food occurs frequently and can result in transfer of pathogens (43, 44). Our results suggest that transfer of bacterial genes across wildlife-livestock interfaces in Nairobi is greatest when livestock are kept at higher densities in low-income settings characterized by low wildlife diversity (rodent interface), when manure is poorly disposed of in less biologically diverse and therefore natural-resource poor habitats (rodent and avian interface), and when household environments are characterized by low diversity avian populations (avian interface). Household affluence represents a range of factors that could impact provision of resources and therefore contact between rodents and livestock; low-income urban settings are commonly associated with high densities of rodents (45) and low biosecurity livestock-keeping practices (46, 47). Wealth might also influence the use of antimicrobials and therefore selection pressures for MGEs associated with antimicrobial resistance. As a breeding ground for arthropods (48), the accumulation of livestock manure within household compounds would also be expected to increase the density of rodents and passerine birds, while forming a bridge for bacterial genes, drug residues, and pathogens to be exchanged between livestock and wildlife. It therefore seems likely that in urban environments devoid of other natural resources, higher densities of livestock and their waste not only stimulate population growth of species that seek their resources [rodents, passerine birds, and bats (27, 43)] but can provide an opportunity for pathogens to cross from wildlife into intermediate hosts, from which humans could then be more easily exposed.

The extent to which humans are exposed to animal-borne pathogens is determined by pathogen pressure within the wildlife and livestock populations with which they coexist and behaviors that can facilitate different routes of transmission (26). More than 1.3 million head of livestock are kept within Nairobi to ensure human food security, and in common with many other cities, these populations are increasing (49, 50). Occupational contact with livestock and their products is a well-documented risk factor for spillover of zoonotic pathogens (51–53), but efforts to characterize the risk that livestock-borne zoonoses pose to human health in urban environments focus almost solely on animal-sourced food value chains, and as such, the risk that livestock pose to their keepers and the broader urban population remains poorly documented (54). Known behavioral risk factors for direct human exposure to zoonoses in urban wildlife populations are restricted to a handful of relatively well-studied pathogens (e.g., hantavirus, *Leptospira* spp., and rabies). With the exception of keeping livestock manure within houses—which increased transmission potential between humans and rodents (in line with previous results in the same study system)—we were unable to evaluate specific behavioral risk factors for sharing of bacterial genes between animals and humans. However, our results indicate that people living at higher densities had consistently greater transmission potential with both wildlife and livestock (reservoir and intermediate hosts). Human density is one of several factors that can determine how quickly and extensively a newly emerged pathogen is able to propagate (55), and this finding therefore suggests that high-density urban human populations should be prioritized in zoonotic EID surveillance efforts.

Our results also suggest that wildlife community structure plays a role in transmission potential between wildlife and people, with people being more central within wildlife networks when inhabiting environments that support a lower diversity of wildlife species. Loss of biodiversity—known as biotic homogenization—generally occurs along gradients of increasing urbanization in the tropics and is associated with the proliferation of synanthropes such as rodents and passerine birds at the expense of other wildlife (27). These species not only live in closer association with people (which may explain that they share more MGEs with people) but are also more competent reservoirs for zoonotic pathogens (34).

### Study Limitations.

We present high-resolution genetic data, collected as part of a detailed epidemiological study and then used to study cross-species transmission potential in a multihost urban system. Urban environments have not been sampled at this scale before, and while this allowed us to draw inference across an urban landscape, our results should be interpreted with the following considerations in mind. Although use of commensal bacteria to infer cross-species transmission potential of other pathogens and its limitations are well established, the validity of extending this approach to populations of their mobile genes is untested [although not without precedent (22). We addressed this by testing the effects of epidemiological parameters on the population structure of MGEs in our dataset and found that *E. coli* belonging to the same sequence type tended to host similar MGE communities. Once the effects of MLST, host taxa, and environmental factors on MGE population structure were removed through variance partitioning, spatial differentiation between MGE communities was negligible, meaning that a hypothesis of MGE population differentiation through isolation by distance could be rejected. This is important because it defines our epidemiological interpretation of transmission potential as it relates to pairwise sharing of genes in our study system—that the number of MGEs shared between *E. coli* is representative of broad transmission chains (which might also be related to common ecological or environmental sources) rather than direct transmission of bacteria between hosts sampled in this study. However, by selecting a single *E. coli* isolate from each host, we only sample a fraction of each host’s *E. coli*–borne MGE diversity. This decision, which was made as a cost-based tradeoff to promote genetic resolution and the number and taxonomic breadth of hosts sampled across the city, limits the statistical power of our study but does not invalidate the results that we report (20). Under-sampling within-host diversity would only be likely to lead to a signal being missed, rather than changes to the positive results that we report in this study.

### Implications for Urban Planning and Public Health Policy.

We find that low biodiversity, coupled with livestock management practices and more densely populated urban environments, promotes sharing of bacterial genes between animals and humans across an urban landscape. Rapid global expansion of urban land use is forecast over the coming decades, and it is therefore crucial that urban development planning and public health policy consider factors underpinning the emergence of infectious diseases. The impacts of losses in biodiversity and human density we document on cross-species transmission potential between wildlife, livestock, and humans suggest that surveillance for zoonotic pathogens should be focused on people and animals who coinhabit densely populated areas within rapidly developing cities. However, while our results indicate characteristics of urban landscapes that promote cross-species transmission of *E. coli* genes, they do not account for pathogen-specific variations in hazard within animal reservoirs and the degree to which these hazards become realized EID risks to people. Governed by host population dynamics, behavioral factors that affect human exposure, immunological variation, and within-host pathogen interactions, these are critically important components of spillover risk that can be influenced by urbanization for animals (22, 56) and humans.

Multidisease studies of urban systems that integrate ecology, sociology, and microbiology to capture how small-scale socioecological and epidemiological conditions must align and multiply for pathogens in animal reservoirs to spillover and amplify in humans would advance us toward a direct assessment of spillover risk for residents of rapidly urbanizing cities. Models generated by these approaches would open the door to more effective toolsets for management-oriented stakeholders and policymakers to pre-empt and address health challenges—whether through more effective forecasting of how urbanization reshapes zoonotic hazards and exposures to guide surveillance and urban planning or frameworks within which to simulate and test the impact of direct interventions on spillover risk. Such studies are required to explore policy interventions aimed at improving health in rapidly urbanizing environments by considering questions such as i) Where should surveillance efforts for early detection of novel pathogens in human populations be deployed? ii) How will future urban social and environmental change (e.g., changing livestock-keeping practices, human demography, deforestation, and loss of biological diversity) influence the likelihood of cross-species transmission? iii) How might policy interventions aimed at promoting biosecurity, biological diversity (e.g., reforestation), and access to green space influence human exposure to zoonotic hazards across a city? Answers to these questions will help policymakers and urban developers make informed decisions that promote the health of people and animals living in urban environments.

## Methodology

### Study Design.

Components of the methods used to collect data presented in this study have been previously described (22, 56). Fecal samples (n = 547) were collected from 57 avian species from 99 households across Nairobi, that were participating in the UrbanZoo project (20). This project, based in Nairobi, Kenya from 2012 to 2017, utilized a landscape genetics approach to understanding the movement and sharing of pathogens in a major developing city. The present study was nested within the “99 household project”—a key component of the UrbanZoo project, which focused on the presence of livestock and wildlife in urban households as a route of zoonotic disease emergence in humans. As such, households were selected with the aim of maximizing the spatial distribution and diversity of livestock-keeping practices across Nairobi and were chosen to capture three main criteria: socioeconomic diversity, population distribution, and livestock-keeping practices. Geospatial mapping data, generated as part of a technical report produced by Institut Français de Recherche en Afrique, were used to identify 17 classes of residential neighborhood in Nairobi based on physical landscape attributes, which were subsequently verified by 817 household questionnaires (57) (*SI Appendix*, Fig. S2). Each of the 17 classes of neighborhood was then ranked by average income and reduced into seven wealth groups. Administrative sublocations were mapped onto each wealth group, identifying a total of 70 possible sublocations, for which dominant wealth groups were calculated by extracting the proportion of the population belonging to each neighborhood class within the sublocation boundaries (*SI Appendix*, Table S2). A total of 33 sublocations were selected to be included in the study, with the number of sublocations belonging to each wealth group chosen proportionately to the population density and the variety of neighborhood classes in each of the seven wealth groups. The final selection of individual sublocations was aimed at maximizing areas with high livestock densities, while ensuring coverage of other neighborhood classes and geographical spread.

For each sublocation, three geographical points were selected at random within the dominant housing type. The order in which sublocations were visited was randomized. Local officials assisted in the recruitment of a household closest to each geographical point to obtain two livestock keeping and one non-livestock-keeping household per sublocation (a total of 99 households, 66 of which kept livestock). Households had to meet strict inclusion criteria of keeping either large ruminants (cattle), large monogastrics (pigs), small ruminants (goats/sheep), small monogastrics (poultry/rabbits), or no livestock species. To ensure an equal sample of both cattle and pig-keeping households, the combination of livestock-keeping households represented in each sublocation was randomized and had to consist of either large ruminant and small monogastric or large monogastric and small ruminant species. For sublocations in which households keeping large ruminant or large monogastric species were absent, a replacement household keeping either small monogastric or small ruminant species was recruited. Sampling of households took place between September 2015 and September 2016.

### Wildlife Trapping and Ecological Surveys.

Two dedicated field teams were responsible for collecting data on humans, livestock, and wildlife in each household, consisting of veterinarians, animal health technicians, and clinicians. Informed consent was obtained from human participants, who were invited to submit a stool sample. Up to 20 rectal swabs were obtained from livestock species present in the household (ensuring that all species were represented in the sample). Rodents and birds were targeted for wildlife sampling. Rodents were trapped using medium-sized (23 cm × 7.5 cm × 9 cm) Sherman live traps (H. B. Sherman Traps Inc., Tallahassee, FL) or Victor lethal traps (Woodstream Corp., Lititz, PA) that were baited with dried fish, placed against walls throughout the household and livestock-keeping facilities, and left in place for three nights. Where possible, traps were set in each household for all trapping nights and checked daily. Mist nets were set at dawn to trap birds, with nets being positioned outside the house and around livestock-keeping facilities. Due to large variation in the size of household compounds, trapping effort (i.e., number of traps/mist nets placed per trapping session) was maintained such that it was proportional to the size of the household compound. The number of wildlife and livestock sampled are presented in electronic *SI Appendix*.

Cross-sectional data were collected on the presence of avian species, and select mammal taxa (rodents, fruit bats, insectivorous bats, nonhuman primates, and small carnivores) in each household compound, from biological sampling activities, ecological surveys, and the household questionnaire. Avian and mammalian taxa were grouped into ecologically relevant functional groups, by their feeding and positional ecology, using the EltonTraits database (58). For birds, the number of different species in each functional group was also calculated in each household. Wildlife biodiversity was estimated from the presence of wildlife species/functional groups within each household. Since we were unable to establish a reliable method of surveying the presence of mammalian species within households, we relied on more easily identifiable mammalian functional groups as a proxy for the diversity of mammals present in each household environment. Wildlife diversity was approximated by adding avian species richness (the total number of avian species recorded in a household) to the number of mammalian functional groups identified as being present in each household.

### Household Questionnaires.

A nominated member of each household completed a questionnaire, detailing i) livestock ownership, management, sourcing, sales, and antimicrobial use, and ii) household composition and socioeconomic data. Abundance (counts) of livestock species and humans were derived from these data for each household. Dividing the total number of livestock and human occupants by household area (meters^2^, as measured using ArcGIS) generated an estimate of livestock and human density for each household. Household composition and socioeconomic data were used to generate wealth and ruralness indices for each household sampled (59). These indices were calculated based on methods used to create the Demographic and Health Surveys (DHS) wealth index, which is derived from a PCA of easily measurable households assets (such as access to water, construction materials, and ownership of livestock) (60). A modification was made to the original set of household assets included in the DHS index to better capture household variation in Nairobi. All field data were recorded using Open Data Kit Collect software (Hartung et al. (61)), on electronic tablets, and uploaded to databases held on servers at the International Livestock Research Institute (ILRI).

### Animal Care and Use and Human Ethics Statement.

The collection of data adhered to the requirements of the ILRI. Wildlife were trapped under the approval of an ILRI Institutional Animal Care and Use Protocol (2015.12). Questionnaire data were collected under ILRI Institutional Research Ethics Committee approval (2015-09), and prior informed consent was gained for each individual participating in the project.

### Land-Use Classification.

Nairobi is characterized by a large variety of land use. Land use comprises the biotic and abiotic niches within which hosts exist and was classified for each household. The boundary of each household compound was drawn in ArcMap, and a 30-m buffer was created around the perimeter of each compound to represent the landscape surrounding it. A buffer of 30 m was chosen to reflect the home range of common urban rodent species (*Mus* and *rattus* spp., estimates of which vary from 1 m to 30 m) (62, 63). Visual classification of land-use types within the compound and buffer area was conducted at 1:500 scale on a 1-m resolution ESRI World Imagery satellite image available in ArcGIS 10.5 (ESRI). Characterization of ecological characteristics along a perimeter around the household compound was considered important because the ecological setting within which the household exists extends beyond the boundaries of the compound. The extent to which this influential area of habitat outside the compound extends is unknown, and as such, it was standardized across study sites. Within the boundary, the areas of nine different land-use types were visually identified and sketched as polygons: water body, wetland, crops, mature trees, shrubs, grassland, bare ground, artificial ground, and rubbish (descriptions for each of these are summarized in *SI Appendix*, Table S3). The total area of classified land-use types at each site was calculated and expressed as proportions. Ecological land-use types (all except bare ground, artificial, and rubbish) were used to calculate Simpson’s diversity index, which considers both habitat richness, and an evenness of abundance among the land-use types present at each site. This index was created to represent the diversity of living (biotic) habitat niches available to wildlife within households and ranged from 1 (maximum heterogeneity) to 0 (only a single category of biotic land use present). All classification was undertaken by J.M.H. who was familiar with the landscape at each site and subsequently ground-truthed by revisiting sites.

### Microbiological Testing.

All swabs and fresh fecal samples were placed in Amies transport media and transported on ice to one of two laboratories (Kenya Medical Research Institute or University of Nairobi). Samples were enriched in buffered peptone water for 24 h and plated onto eosin methylene blue agar (EMBA). Plates were incubated for 24 h at 37 °C, after which five colonies were selected from each EMBA plate. After a further subculture on EMBA to purify the isolates, the pure isolates were subcultured on Müller-Hinton agar and archived at −80 °C in cryovials containing Soy broth supplemented with 15% glycerol.

### Next-Generation Sequencing.

A single colony was picked from each original sample (referred to as an isolate) and biochemical tests (triple sugar iron agar, Simmon’s citrate agar, and motility-indole-lysine media) were run for identification as *E. coli*. DNA was extracted from bacterial isolates using commercial kits (Purelink® Genomic DNA Mini Kit, Invitrogen, Life Technologies, Carlsbad, California) and transported under license to The Wellcome Trust Centre for Human Genetics, Oxford, United Kingdom. WGS was carried out at the Wellcome Trust Centre for Human Genetics on the Illumina HiSeq 2500 platform. One hundred fifty base-pair paired-end reads were generated, and short-read WGS data were preprocessed using an automated protocol developed by the Modernising Medical Microbiology Oxford Group to i) perform standard quality control checks using fastQC (https://www.bioinformatics.babraham.ac.uk/projects/fastqc/) with default settings; ii) trim reads to remove remnant adaptor sequences using bbduk (64) (parameters: minoverlap = 12, k = 19, mink = 12, hdist = 1, ktrim = r) and iii) perform a Kraken (65) speciation analysis against with an in-house database of bacterial reads downloaded from the NCBI sequence read archive (www.ncbi.nlm.nih.gov/sra/), with an automated step for removal of contaminant (nonbacterial) reads. De novo assembly was performed using SPAdes v3.6 (66) (parameters: --careful, -t 1, --phred-offset 33). The assemblies were run through the batch upload mode of the Centre for Genetic Epidemiology web interface hosted by the Technical University of Denmark (https://www.genomicepidemiology.org/services/) which performs speciation analysis (67), multilocus sequence typing (MLST) (68), detection of resistance genes (69), and detection of virulence genes (70). The threshold of AMR gene detection was set to 90% identity and 60% coverage, as this is shown to be the optimal threshold for this method. A 60% coverage threshold was used to ensure that AMR genes spread over two contigs, and/or located on the edge of the contig, were not missed (69). Virulence genes were identified using VirulenceFinder with 90% minimum match and 60% minimum length. Samples deemed as non–*E. coli* on the basis of the speciation analysis with kmerFinder (71) in the Centre for Genetic Epidemiology pipeline were excluded from further analysis. Potentially mixed *E. coli* samples were identified as those with an unusually large assembly size [greater than 6 megabases (Mb)] and were removed from the dataset.

To address the fact that genes comobilized on the same MGE might not represent independent acquisition events without having access to long-read sequencing (which would enable identification of the location of genes on plasmids), we combined all pairs of genes with 100% co-occurrence (e.g., bfpA and perA).

### Statistical Analyses.

All statistical analyses were conducted using R v3.3.2 (72). Spatial structure in the dataset was represented using distance-based Moran’s eigenvector maps—a powerful multivariate approach to model spatial structure in a response variable, which can be partitioned at broad, medium, and fine spatial scales (73, 74). Further details of how we dealt with missing data, data exploration, and statistical models (distributions, choice of fixed and random effects, implementation, and model selection procedures) are given in the *SI Appendix*.

### Canonical Analysis.

A distance-based redundancy analysis (db-RDA) was used to test hypotheses related to isolation by distance vs. isolation by environment. In this method, the response variable is represented as a distance matrix (here, dissimilarities in MGE assemblages between hosts were represented by the Jaccard distance coefficient) and input into a Principal Coordinate Analysis (PCoA). The resulting PCoA eigenvectors represent the dissimilarities in a Euclidean space and can be input as the response variables in a standard RDA and regressed against the set of explanatory variables (75). Model selection was performed using forward selection with a double-stopping criterion, which aims to maximize the adjusted coefficient of multiple determination (adjusted R^2^) at each step (76). The double-stopping criterion addresses two problems typically associated with forward selection methods - high type I error and including too many explanatory variables in the model. This is achieved by running a global test on the full model first, and only progressing if that test is significant by permutation, and stopping the selection process if a candidate variable is deemed non-significant, or if it brings the adjusted R^2^ of the model over the value of the adjusted R^2^ of the global model. Significance testing of parsimonious models was undertaken using nonparametric permutation tests, in which random permutations of the response and explanatory variables are used to create a “null distribution” that is compared using test statistics (the pseudo-F-value) against the unpermuted input data. PCoA eigenvectors representing dissimilarities in MGE assemblages between hosts were treated as the response variable, and explanatory variables included i) the spatial relationships between each host at the time of sampling; ii) MLST of each E. coli isolate; iii) the host taxa from which each bacterial isolate was cultured, and iv) the ecological and anthropogenic characteristics of each household. Permutations were restricted within sublocations to account for correlation between samples collected from the same household. The influence of each set of explanatory variables on dissimilarity of MGE communities between hosts was tested by partitioning the variance of spatial, environmental, and genetic determinants within the db-RDA model (77). Statistical significance of each fraction with respect to all others was tested using seperate db-RDAs and ANOVA. All canonical analysis was conducted in the R package “vegan” (78).

### Network Analysis.

Bipartite and unipartite TPNs were created using the R-package iGraph, following the approaches developed by Pilosof et al. (16). In the projected unipartite networks, each edge linking two nodes was weighted by the number of MGEs that each node had in common. While edge weights in mTPNs are often represented by measures of beta diversity (such as the Jaccard dissimilarity index) to avoid introducing bias due to variation in detection effort for parasites within hosts, the process of detecting *E. coli*-borne MGEs through WGS in this study was unbiased in the sense that detection of a particular gene is not presupposed. A single-layer TPN with an ergm was used to model network structure and the likelihood of MGEs being shared within and between host taxa across Nairobi. The initial ergm consisted of the structural edges term, which represents the probability of an edge being formed in the graph. Categorical node-level covariates host taxa (avian, rodent, human, and livestock) and household were added to the model, along with an edge-level covariate representing whether two nodes shared the same MLST of *E. coli*. Household membership was included to control for spatial dependency at the household level between nodes, while pairwise sharing of sequence type incorporated the effects of *E. coli* genetic structure into the model. A best-fit model was selected which contained the fewest covariates while maintaining the lowest AIC value. Goodness of fit was determined by comparing metrics of networks simulated using the ergm model to the observed network metrics. Models were fit using Maximum Penalized Likelihood Estimation in the R package *ergm*.

Multilayer TPNs were created to examine connectivity between hosts across interfaces and consisted of unipartite networks which represented the connectivity of an individual host with all hosts belonging to a different taxonomic group (as described in the main text and represented in Fig. 1). We followed others in using node centrality as a proxy for the importance of individual hosts within each multilayer network of shared MGEs. Seven degree- and eigenvalue-derived centrality measures and six distance-based measures were calculated for each mTPN (for further details, see *SI Appendix*, Table S1). These included measures based on Degree and EC, which in epidemiological terms represent the importance of each node based on its immediate risk of transmitting or receiving shared elements to other nodes in the network, and Closeness and Betweenness centrality, which measure each node’s proximity to other nodes in the network through shared elements, and the extent to which each node links otherwise sparsely connected parts of the network. Due to the large number of available centrality metrics that either represent a distinct measure of direct pathogen sharing between hosts (degree and eigenvector-based measures) or indirect sharing of pathogens between hosts (distance-based measures), we followed Wardeh et al. (31) in using PCA (in the R package FactoMineR) to select the centrality measures that best represented network structure. In this way, each host of a particular taxonomic group (e.g., humans) was assigned a set of relevant centrality measures to represent their propensity to share MGEs (transmission potential) with all of the hosts belonging to a different taxonomic group (e.g., rodents).

To determine factors that promote transmission potential across taxonomic interfaces, host centrality was regressed against a set of anthropological and ecological explanatory variables using GAMs, on the assumption that our dataset contained nonlinear relationships. Explanatory variables were selected a priori on the basis of existing knowledge of factors shaping transmission in rapidly developing urban landscapes. To account for spatial dependency within the dataset, the coordinates of the site at which each host was sampled were modeled as a Gaussian process smooth. Since we expected MLST to play a major role in patterns of MGE occurrence, the relationship between bacterial population structure and MGE diversity was also accounted for by including the *E. coli* MLST for each host as a random effect. The proportion of deviance explained by individual terms in each model was calculated as (*D_i_*−*D_F_*)/*D_0_*, where *D_i_* is the deviance of model*_i_* (with the individual term removed), *D_F_* is the deviance of the full model, and *D_0_* is the deviance of an intercept-only model. The smoothing parameters from the best model were used throughout the set of models used to calculate deviance explained. Because model terms were not exactly orthogonal, deviance explained did not sum to the total deviance explained by each model. All models were fit using restricted maximum likelihood in the R package mgcv (79), and model selection was performed using the automated shrinkage via double penalty approach. Model validity was checked using standard methods included in the package mgcv (including examination of concurvity, the basis dimensions used for smoothing terms, and diagnostic plots).

### Reduced Dataset without Cosmopolitan Bacterial Genes.

To account for variation in the distribution of MGEs between different host taxa as a potential source of bias when calculating network centrality, MGEs that appeared in similar proportions across all host taxonomic groups were removed. Two such “reduced” datasets were created—i) a less stringent classification in which select MGEs that were found at a high frequency across taxonomic groups were removed and ii) a more stringent and standardized classification, in which MGEs for which the highest proportion found in any one host taxa was less than 36% (when compared across other host taxa) were removed, and subsequently, all MGEs with a variance of less than 0.01 between the relative proportion found in each of the four host taxa (wild birds, rodents, livestock, and humans) were removed. Analysis of mTPNs (using centrality measures and GAMs, as described above) was repeated using each of these datasets (*SI Appendix*, Table S4).

## Supplementary Material

Appendix 01 (PDF)

## Data Availability

Data are available via an open access repository held by the University of Liverpool (https://doi.org/10.17638/datacat.liverpool.ac.uk/2236) (80).
