# Supplementary material for: Epidemiological connectivity between humans and animals across an urban landscape
Source: Proc Natl Acad Sci U S A. 2023 Jul 14;120(29):e2218860120. doi: 10.1073/pnas.2218860120 (PMC10629570; doi:10.1073/pnas.2218860120)
Supplement: Supplementary file 1 — Appendix 01 (PDF) [file pnas.2218860120.sapp.pdf]

PNAS

Supporting Information for

Epidemiological connectivity between humans and animals across an urban landscape

James M. Hassell[1,2,3($)], Dishon M. Muloi[4,5,6], Kimberly L. VanderWaal[7], Melissa J Ward[4,8,9], Judy Bettridge[3,5], Nduhiu Gitahi[10], Tom Ouko[11], Titus Imboma[12], James Akoko[5], Maurice Karani[5], Patrick Muinde[5], Yukiko Nakamura[13], Lorren Alumasa[5], Erin Furmaga[14], Titus Kaitho[15], Fredrick Amanya[5], Allan Ogendo[5], Francesco Fava[5,16], Bryan A. Wee[4], Hang Phan[8], John Kiiru[11], Erastus Kang'ethe[10], Sam Kariuki[11], Timothy Robinson[17], Michael Begon[3], Mark E. J. Woolhouse[4,6(†)], Eric M. Fèvre[3,5($,†)]

1. Global Health Program, Smithsonian's National Zoo and Conservation Biology Institute, Washington DC, US
2. Department of Epidemiology of Microbial Diseases, Yale School of Public Health, Connecticut, US
3. Institute of Infection, Veterinary and Ecological Sciences, University of Liverpool, Neston, UK
4. Usher Institute, University of Edinburgh, Edinburgh, UK
5. International Livestock Research Institute, Nairobi, Kenya
6. Centre for Immunity, Infection and Evolution, University of Edinburgh, Edinburgh, UK
7. Department of Veterinary Population Medicine, College of Veterinary Medicine, University of Minnesota, St. Paul, US
8. Nuffield Department of Clinical Medicine, University of Oxford, Oxford, UK
9. Faculty of Medicine, University of Southampton, UK
10. University of Nairobi, Nairobi, Kenya
11. Kenya Medical Research Institute, Nairobi, Kenya
12. National Museums of Kenya, Nairobi, Kenya
13. Faculty of Veterinary Medicine, Hokkaido University, Japan
14. Department of Epidemiology, Columbia University, New York, US
15. Veterinary Services Department, Kenya Wildlife Service, Kenya
16. Department of Environmental Science and Policy, Università degli Studi di Milano, Milan, Italy
17. Food and Agriculture Organization of the United Nations, Rome, Italy

James M. Hassell
Email: hassell.jm@gmail.com

This PDF file includes:

Figures S1 to S2
Tables S1 to S4
References

Fig. S1. Variables included in this study, relative to a hypothetical framework for urban animal-to-human pathogen spillover risk.

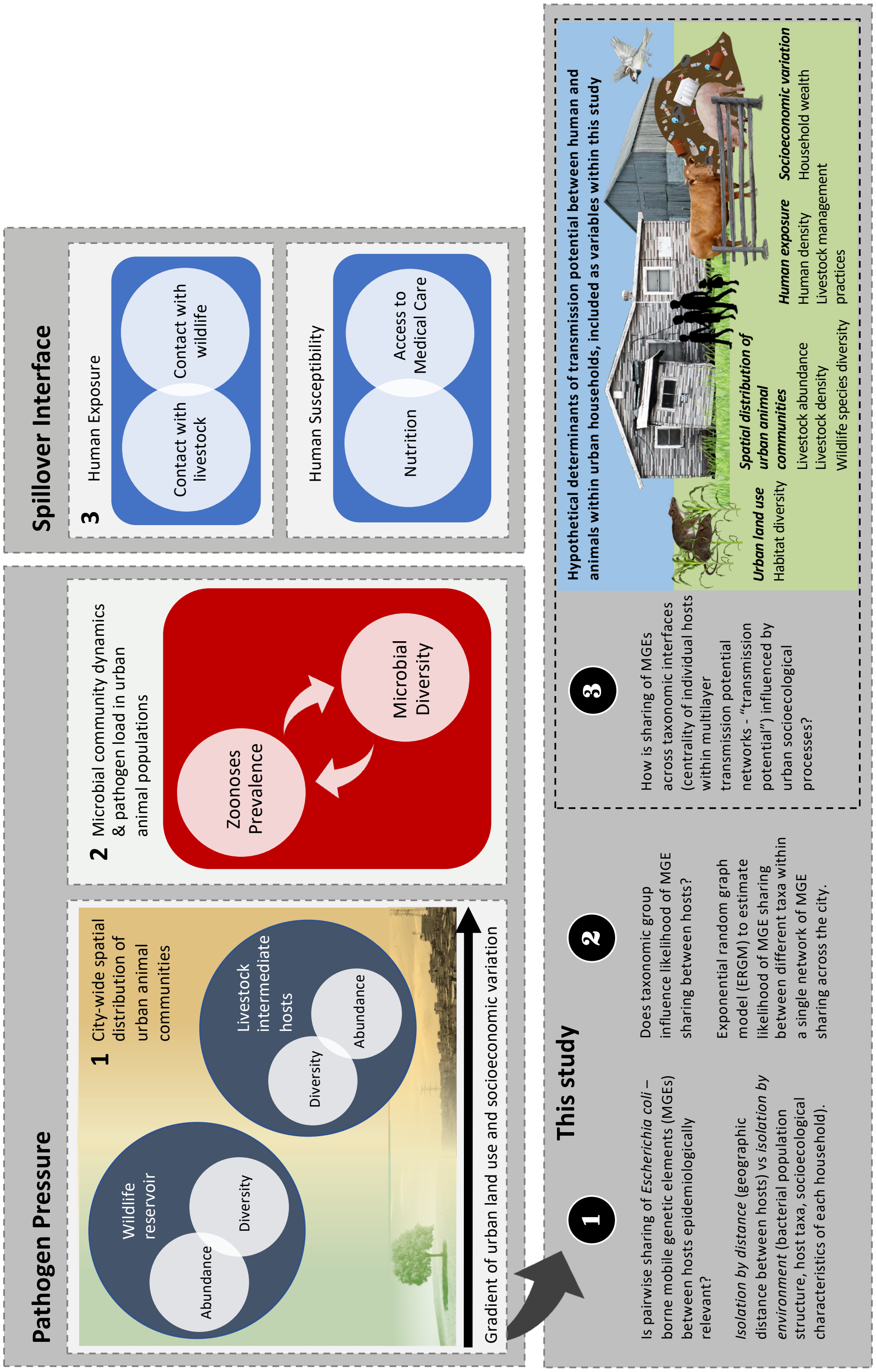

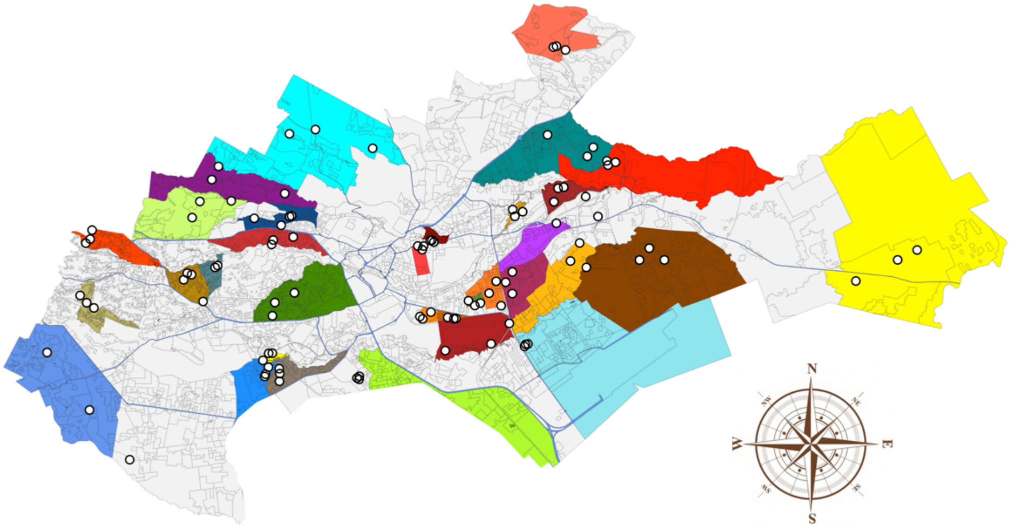

Fig. S2. Map of the 100 households (white dots) and 33 sublocations (individually coloured areas) in Nairobi selected for inclusion in the study. Areas of the city in grey represent those not samples.

Measure	Assumption/Interpretation	Mean contribution towards first principle component
**Degree and eigenvector-base measures**		
Degree centrality (DC)	Degree quantifies the immediate risk of a host in spreading or receiving MGEs. Hosts with high degree may either: 1) pose high risk of MGE transmission to many other host species, or 2) be at high risk of contracting MGEs from many other hosts.	4.60
Opsahl degree centrality ODC)	Incorporates both the overall weights of the edges (number of shared MGEs) and the number of links to neighbouring hosts.	10.97
Eigenvector centrality (EC)	Eigenvector quantifies the immediate risk of a host in spreading or receiving MGEs from, or to, other hosts that themselves have a high risk of transmitting MGEs to many other hosts.	10.34
Weighted eigenvector centrality (WEC)	Weighted variation of eigenvector centrality, by which edges encompassing larger number of MGEs are considered more important than those containing fewer MGEs.	10.35
Strength centrality (SC)	Hosts with high strength centrality values either: 1) share many MGEs with few hosts, or 2) share fewer MGEs with many hosts.	10.71
Page rank/Weighted page rank centrality (PRC/WPRC)	PageRank is an adjustment of Katz centrality. Katz centrality quantifies the importance of a host within a network by measuring the number of immediate neighbours the host has, and also all other hosts reachable through these immediate neighbours. In PageRank, the more links a host has with other hosts (in terms of shared MGEs), the more important it is perceived.	5.36
**Distance based measures**		
Closeness centrality (CC)	Closeness is the inverse sum of shortest distances to all other host species from a focal host.	5.16
Weighted closeness centralitry (WCC)	Weighted variation of closeness, by which edges encompassing larger number of MGEs are considered shorter than those containing fewer MGEs.	9.36
Opsahl closeness centrality (OCC)	Extends the shortest path algorithm by taking into consideration the number of intermediary hosts. ODC considers both the number of intermediary hosts (nodes) and the edges' weights (number of MGEs).	10.57
Betweenness centrality (BC)	Betweenness centrality quantifies the number of times a host species acts as a bridge along the shortest path between two other hosts. It thus describes the importance of that node as an intermediary between different parts of the network. Hosts with betweenness > 0 act as bridges, connecting one part of a network to another that would otherwise be sparsely connected or not connected at all.	6.14
Weighted betweenness centrality (WBC)	Weighted variation of betweenness. The more MGEs that are shared between two nodes, the stronger of the flow between them.	4.52
Opsahl betweenness centrality (OBC)	Extends betweenness by combining both the number of intermediary hosts (nodes) and the edges' weights (number of shared MGEs).	0.55

Table S1. Adapted from Wardeh et al(1). Measures of node centrality considered for use within this study, and their mean contribution (%) to the first principal component in a principal component analysis performed on each unipartite network.

Characteristics of physical neighbourhood classes identified by IFRA study (adapted from Ledant *et al.* [1])				Urban Zoo Project re-classification		
Tree cover	Defining characteristics	Neighbourhood description (housing type)	Average income	Wealth group	Possible sub-locations	
> 13.5%	Detached housing with intense tree cover	Detached housing on very large plots *(>3000 m²)*	39,890	1	8	3
		Detached housing on large plots *(400 - 3000 m²)*	22,462	2	8	4
	Attached and semi-detached housing	Attached housing on medium plots *(<400 m²)* with important tree cover	22,084	2		
3% < 13.5%	Apartment building	Apartment buildings *with gated space*	22,084	2		
	Attached and semi-detached housing	Higher standing row houses *(plot size > 190 m²)*	13,352	3	5	3
		Lower standing row houses *(plot size < 190 m²)*	6,153	4	3	3
<3%	Roof cover >50% tiles	Lower standing *apartment buildings*	6,153	4		
		New areas of dense *single housing* development	3,855	5	9	5
	Roof cover > 40% concrete	High density multi-storey buildings	3,855	5		
3% < 13.5%	Apartment building	Apartment buildings *with open access*	3,855	5		
	Peripheral areas	Peripheral areas with residential component *(mainly residential)*	3,855	5		
		Peripheral areas with rural component *(presence of agriculture)*	2,165	6	24	11
	Collective housing	Community housing *with gated space*	2,165	6		
		Community housing *with open access*	2,165	6		
<3%	Roof cover >85% corrugated iron sheets	New areas of low quality housing *(built-up area <37%)*	2,165	6		
		High density planned low quality housing *(built-up area <37% AND public space >20%)*	2,165	6		
		High density unplanned low quality housing (slums) *(built-up area <37% AND public space <20%)*	1,301	7	13	4

Table S2. The seven wealth groups used by the UrbanZoo Project, and the number of sub-locations with a dominant wealth group identified and selected in the Nairobi municipality.

Land Use Class	Explanation
Water-body (environmental, biotic)	Natural water body (flowing or non-flowing)
Wetland (environmental, biotic)	Open wetland – reeds/rushes
Cropland (anthropogenic, biotic)	Row cops or other herbaceous crops (e.g. maize, coffee etc.)
Trees (environmental, biotic)	Trees (single or multiple), as determined by presence of a clear crown and evidence of shadow cast on ground (includes plantations of trees for commercial purposes [e.g. fruit])
Shrubs (environmental, biotic)	Shrubs, where able to distinguish from grassland and trees
Grassland (environmental, biotic)	Grass, pasture, herbaceous rangeland or bare-ground not serving a human purpose.
Bare ground (anthropogenic, abiotic)	Heavily compacted soil, serving human purpose (e.g. dirt road, playground)
Artificial (anthropogenic, abiotic)	Synthetic, man-made surface or object (including water-bodies) (e.g. tarmac road, cement, roof, swimming pool, water tank)
Rubbish (anthropogenic, abiotic)	Accumulation of human-derived waste

Table S3. Land use classifications

		Model 1				Model 2 - filtered for singletons/doubletones and very common genes				Model 3 - variance above 0.009			
Network	Variable	Effective degrees of freedom	Chisqstatistic	P.value	Relative deviance	Effective degrees of freedom	Chisqstatistic	P.value	Relative deviance	Effective degrees of freedom	Chisqstatistic	P.value	Relative deviance
avian-avian_EC	s(avian diversity)	0.7	0.35	0.06	0.02	0.7	0.35	0.06	0.02				
	s(human density)	0	0	1	0.00	0	0	1	0.00				
	s(livestock density)	0.37	0.08	0.198	0.01	0.37	0.08	0.198	0.01				
	s(habitat diversity)	0.79	0.58	0.024	0.02	0.79	0.58	0.024	0.02				
	s(MLST)	34.27	0.41	0.004	0.75	34.28	0.41	0.004	0.75				
	s(wealth)	0	0	0.784	0.00	0	0	0.783	0.00				
	s(x,y)	1.07	0.1	0.083	0.03	1.07	0.1	0.083	0.03				
avian-avian_ODC	s(avian diveristy)	1.25	0.57	0.067	0.02	1.25	0.57	0.067	0.02				
	s(human density)	0	0	0.985	0.00	0	0	0.985	0.00				
	s(livestock density)	0	0	0.604	0.00	0	0	0.604	0.00				
	s(habitat diversity)	0.85	1.05	0.008	0.02	0.85	1.05	0.008	0.02				
	s(MLST)	50.85	0.77	<0.001	0.85	50.86	0.77	<0.001	0.85				
	s(wealth)	0	0	0.589	0.00	0	0	0.589	0.00				
	s(x,y)	0.64	0.04	0.195	0.01	0.64	0.04	0.195	0.01				
avian-avian_OCC	s(avian diveristy)	0.62	0.28	0.099	0.01	0.62	0.28	0.098	0.01				
	s(human density)	0	0	1	0.00	0	0	1	0.00				
	s(livestock density)	0	0	0.515	0.00	0	0	0.516	0.00				
	s(habitat diversity)	0.83	0.92	0.014	0.01	0.83	0.92	0.014	0.01				
	s(MLST)	52.34	0.8	<0.001	0.84	52.35	0.8	<0.001	0.84				
	s(wealth)	0	0	0.682	0.00	0	0	0.679	0.00				
	s(x,y)	1.23	0.15	0.066	0.02	1.23	0.15	0.066	0.02				
avian-livestock_EC	s(avian diveristy)	0.67	0.29	0.072	0.02	0.68	0.3	0.072	0.02				
	s(livestock density)	0.16	0.02	0.265	0.00	0.18	0.03	0.262	0.00				
	s(habitat diversity)	0.82	0.69	0.014	0.03	0.82	0.69	0.014	0.03				
	s(MLST)	31.25	0.36	0.01	0.73	31.09	0.35	0.011	0.73				
	s(wealth)	0	0	0.802	0.00	0	0	0.803	0.00				
	s(x,y)	1.1	0.11	0.073	0.03	1.1	0.11	0.073	0.03				
avian-livestock_ODC	s(avian diveristy)	0.77	0.31	0.095	0.01	0.77	0.31	0.095	0.01				
	s(livestock density)	0	0	0.672	0.00	0	0	0.672	0.00				
	s(habitat diversity)	0.85	1	0.008	0.02	0.85	1	0.008	0.02				
	s(MLST)	46.92	0.65	<0.001	0.82	46.94	0.65	<0.001	0.82	Models did not converge			
	s(wealth)	0	0	0.663	0.00	0	0	0.663	0.00				
	s(x,y)	1	0.1	0.101	0.02	1	0.1	0.101	0.02				
avian-livestock_OCC	s(avian diveristy)	0.58	0.21	0.117	0.01	0.57	0.21	0.118	0.01				
	s(livestock density)	0	0	0.716	0.00	0	0	0.691	0.00				
	s(habitat diversity)	0.85	1.02	0.007	0.02	0.85	1.02	0.007	0.02				
	s(MLST)	44.26	0.59	<0.001	0.81	44.39	0.6	<0.001	0.81				
	s(wealth)	0	0	0.665	0.00	0	0	0.671	0.00				
	s(x,y)	1.09	0.13	0.073	0.02	1.1	0.13	0.073	0.02				
aviran-rodent_EC	s(human density)	0	0	1	0.00	0	0	1	0.00				
	s(livestock density)	0.31	0.06	0.219	0.00	0.31	0.06	0.219	0.00				
	s(habitat diversity)	0.83	0.9	0.011	0.02	0.83	0.9	0.011	0.02				
	s(MLST)	45.9	0.66	<0.001	0.82	45.89	0.65	<0.001	0.82				
	s(wealth)	0	0	0.621	0.00	0	0	0.67	0.00				
	s(x,y)	1.08	0.12	0.083	0.02	1.08	0.12	0.082	0.02				
	s(wildlife diversity)	0.43	0.11	0.171	0.01	0.44	0.11	0.17	0.01				
avian-rodent_ODC	s(human density)	0	0	1	0.00	0	0	1	0.00				
	s(livestock density)	0	0	0.693	0.00	0	0	0.696	0.00				
	s(habitat diversity)	0.86	1.31	0.005	0.02	0.86	1.31	0.005	0.02				
	s(MLST)	56.2	0.96	<0.001	0.86	56.2	0.96	<0.001	0.86				
	s(wealth)	0	0	0.492	0.00	0	0	0.492	0.00				
	s(x,y)	0.59	0.03	0.225	0.01	0.59	0.03	0.224	0.01				
	s(wildlife diversity)	1.52	0.78	0.06	0.02	1.53	0.79	0.059	0.02				
avian-rodent_OCC	s(human density)	0	0	1	0.00	0	0	1	0.00				
	s(livestock density)	0	0	0.572	0.00	0	0	0.426	0.00				
	s(habitat diversity)	0.84	1.21	0.01	0.01	0.84	1.21	0.01	0.01				
	s(MLST)	61.09	1.1	<0.001	0.88	61.06	1.1	<0.001	0.88				
	s(wealth)	0	0	0.475	0.00	0	0	0.464	0.00				
	s(x,y)	1.2	0.16	0.071	0.02	1.17	0.16	0.071	0.02				
	s(wildlife diversity)	0.35	0.08	0.204	0.00	0.35	0.08	0.205	0.00				
human-avian_EC	s(avian diveristy)	0.78	0.74	0.027	0.01	0.78	0.73	0.027	0.01	0.91	0.26	0.081	0.03
	s(human density)	0.88	1.83	0.003	0.02	0.88	1.84	0.003	0.02	0.85	0.85	0.006	0.04
	s(livestock density)	0	0	0.667	0	0	0	0.667	0.00	0.65	0.14	0.172	0.01
	s(habitat diversity)	0	0	1	0	0	0	1	0.00	0.34	0.07	0.189	0.01
	s(MLST)	66.45	1.57	<0.001	0.85	66.42	1.57	<0.001	0.85	27.65	0.49	0.001	0.72
	s(wealth)	0	0	1	0	0	0	1	0.00	0	0	0.802	0
	s(x,y)	1.59	0.41	0.017	0.02	1.59	0.41	0.017	0.02	1.31	0.17	0.038	0.04
human-avian_OCC	s(avian diveristy)	0.7	0.46	0.063	0.01	0.69	0.46	0.064	0.01	0	0	0.556	0
	s(human density)	0.89	2.01	0.002	0.01	0.89	2.01	0.002	0.01	0.9	1.79	0.001	0.04
	s(livestock density)	0	0	0.635	0	0	0	0.622	0.00	0.79	0.23	0.157	0.01
	s(habitat diversity)	0	0	1	0	0	0	1	0.00	0	0	0.403	0
	s(MLST)	70.32	1.83	<0.001	0.87	70.27	1.82	<0.001	0.87	42.74	1	<0.001	0.82
	s(wealth)	0	0	1	0	0	0	1	0.00	0	0	1	0
	s(x,y)	1.62	0.5	0.012	0.02	1.62	0.49	0.012	0.02	0.9	0.08	0.133	0.01
human-avian_ODC	s(avian diveristy)	0.6	0.25	0.104	0.01	0.6	0.24	0.104	0.01	0	0	0.341	0
	s(human density)	0.88	1.52	0.003	0.02	0.88	1.52	0.003	0.02	0.92	1.95	<0.001	0.06
	s(livestock density)	0	0	1	0	0	0	1	0.00	0.61	0.13	0.193	0.01
	s(habitat diversity)	0	0	1	0	0	0	1	0.00	0	0	0.537	0
	s(MLST)	60.35	1.3	<0.001	0.88	60.34	1.3	<0.001	0.88	35.89	0.74	<0.001	0.78
	s(wealth)	0	0	0.878	0	0	0	0.882	0.00	0	0	0.727	0
	s(x,y)	1.5	0.31	0.025	0.03	1.5	0.31	0.025	0.03	1.25	0.17	0.047	0.03
human-livestock_EC	s(human density)	0.94	4.41	<0.001	0.04	0.94	4.4	<0.001	0.04	0.86	3.14	0.008	0.01
	s(livestock density)	0	0	0.463	0	0	0	0.459	0.00	0	0	0.775	0
	s(MLST)	68.62	1.68	<0.001	0.88	68.47	1.67	<0.001	0.88	73.56	4.67	<0.001	0.97
	s(wealth)	0	0	1	0	0	0	1	0.00	0	0	0.794	0
	s(x,y)	1.29	0.21	0.076	0.01	1.28	0.22	0.077	0.01	0	0	1	0
human-livestock_OCC	s(human density)	0.94	4.33	<0.001	0.04	0.94	4.34	<0.001	0.04	0.91	2.47	<0.001	0.03
	s(livestock density)	0	0	0.501	0	0	0	0.5	0.00	0.92	0.23	0.27	0.01
	s(MLST)	70.2	1.77	<0.001	0.89	70.29	1.78	<0.001	0.89	53.41	1.62	<0.001	0.88
	s(wealth)	0	0	0.859	0	0	0	0.856	0.00	0	0	1	0
	s(x,y)	1.36	0.25	0.057	0.01	1.36	0.25	0.056	0.02	0.1	0	0.327	0
human-livestock_ODC	s(human density)	0.93	3.29	<0.001	0.04	0.94	4.34	<0.001	0.04	0.94	4.05	<0.001	0.05
	s(livestock density)	0	0	0.756	0	0	0	0.5	0.00	0	0	0.508	0
	s(MLST)	64.25	1.46	<0.001	0.89	70.29	1.78	<0.001	0.89	50.99	1.52	<0.001	0.85
	s(wealth)	0	0	0.629	0	0	0	0.856	0.00	0	0	1	0
	s(x,y)	1.33	0.22	0.058	0.02	1.36	0.25	0.056	0.01	0.69	0.05	0.182	0.01
human-rodent_EC	s(human density)	0.87	1.78	0.005	0.01	0.87	1.78	0.005	0.01	0.7	0.56	0.053	0.01
	s(livestock density)	0	0	0.747	0	0	0	0.748	0.00	0.54	0.33	0.136	0
	s(habitat diversity)	0	0	1	0	0	0	1	0.00	0	0	0.926	0
	s(MLST)	73.85	2.05	<0.001	0.87	73.84	2.05	<0.001	0.87	60.06	2.2	<0.001	0.91
	s(wealth)	0	0	1	0	0	0	1	0.00	0.46	0.18	0.15	0
	s(wildlife diversity)	0.8	0.9	0.022	0.01	0.8	0.9	0.022	0.01	0	0	1	0
	s(x,y)	1.62	0.5	0.016	0.02	1.62	0.5	0.016	0.02	0	0	0.883	0
human-rodent_ODC	s(human density)	0.85	1.4	0.007	0.01	0.85	1.4	0.007	0.01	0.91	2.42	<0.001	0.03
	s(livestock density)	0	0	1	0	0	0	1	0.00	0.53	0.26	0.147	0
	s(habitat diversity)	0	0	1	0	0	0	1	0.00	0	0	0.84	0
	s(MLST)	67.82	1.69	<0.001	0.89	67.81	1.69	<0.001	0.89	52.39	1.59	<0.001	0.87
	s(wealth)	0	0	0.905	0	0	0	0.903	0.00	0	0	0.576	0
	s(wildlife diversity)	0.66	0.37	0.073	0.01	0.66	0.37	0.073	0.01	0	0	0.974	0
	s(x,y)	1.54	0.36	0.025	0.02	1.54	0.36	0.025	0.02	0	0	0.819	0
livestock-avian_EC	s(avian diveristy)	1.33	1.5	0.002	0.03	1.33	1.49	0.002	0.03	1.99	2.48	<0.001	0.06
	s(livestock density)	0	0	0.575	0	0	0	0.583	0.00	0	0	0.744	0
	s(habitat diversity)	0	0	1	0	0	0	1	0.00	0	0	0.995	0
	s(MLST)	83.02	0.8	<0.001	0.91	82.99	0.8	<0.001	0.91	62.5	0.8	<0.001	0.88
	s(wealth)	0.64	0.29	0.092	0	0.64	0.29	0.092	0.00	0	0	0.352	0
	s(x,y)	0	0	0.629	0	0	0	0.629	0.00	0	0	0.89	0
	s(avian diveristy)	1.09	1.16	0.009	0.02	1.08	1.15	0.009	0.02	1.49	0.87	0.029	0.03

livestock-avian_OCC	s(livestock density)	0	0	0.746	0	0	0	0.849	0.00	0	0	0.702	0
	s(habitat diversity)	0	0	0.736	0	0	0	0.734	0.00	0	0	0.937	0
	s(MLST)	100.2	1.17	<0.001	0.94	100.17	1.17	<0.001	0.94	63.01	0.74	<0.001	0.94
	s(wealth)	0.68	0.39	0.075	0	0.68	0.39	0.075	0.00	0	0	0.662	0
	s(x,y)	0	0	0.418	0	0	0	0.428	0.00	0	0	0.388	0
livestock-avian_ODC	s(avian diveristy)	0.73	0.48	0.048	0.01	0.73	0.48	0.048	0.01	1.83	1.61	0.004	0.05
	s(livestock density)	0	0	0.609	0	0	0	0.613	0.00	0	0	0.565	0
	s(habitat diversity)	0	0	0.907	0	0	0	0.894	0.00	0	0	0.824	0
	s(MLST)	92.13	1.09	<0.001	0.95	92.11	1.09	<0.001	0.95	60.35	0.74	<0.001	0.9
	s(wealth)	0.77	0.58	0.036	0	0.76	0.58	0.036	0.00	0	0	0.558	0
	s(x,y)	0	0	0.387	0	0	0	0.389	0.00	0	0	0.572	0
livestock-rodent_EC	s(habitat diversity)	0	0	1	0	0	0	1	0.00	0	0	0.378	0
	s(ST)	92.7	1.07	<0.001	0.92	92.71	1.07	<0.001	0.92	95.49	2	<0.001	0.97
	s(wealth)	0.69	0.39	0.064	0	0.69	0.39	0.064	0.00	0.69	0.66	0.065	0
	s(x,y)	0	0	0.601	0	0	0	0.601	0.00	0.06	0	0.341	0
	te(livestock density,wildlif	2.35	0.37	0.032	0.02	2.35	0.37	0.032	0.02	0.67	0.04	0.311	0
livestock-rodent_OCC	s(habitat diversity)	0	0	0.751	0	0	0	0.755	0.00	0	0	0.438	0
	s(MLST)	113.3	1.61	<0.001	0.95	113.3	1.61	<0.001	0.95	43.57	0.49	<0.001	0.93
	s(wealth)	0.72	0.54	0.049	0	0.72	0.53	0.049	0.00	0	0	1	0
	s(x,y)	0.05	0	0.338	0	0.06	0	0.337	0.00	0	0	0.408	0
	te(livestock density,wildlif	1.35	0.24	0.061	0.01	1.35	0.25	0.061	0.01	2.01	0.23	0.087	0.04
livestock-rodent_ODC	s(livestock density)	0	0	0.672	0	0	0	0.764	0.00	0	0	0.34	0
	s(habitat diversity)	0	0	0.987	0	0	0	0.959	0.00	0	0	0.488	0
	s(MLST)	101.17	1.38	<0.001	0.95	101.16	1.38	<0.001	0.95	72.05	1.13	<0.001	0.97
	s(wealth)	0.72	0.48	0.054	0	0.72	0.48	0.054	0.00	0	0	0.448	0
	s(wildlife diversity)	0.74	0.55	0.042	0.01	0.74	0.55	0.01	0.00	0.39	0.09	0.193	0
	s(x,y)	0	0	0.424	0	0	0	0.423	0.00	1.05	0.07	0.208	0.01

Table S4. Since mobile genetic elements (MGEs) appear at different frequencies in our dataset, it is likely that certain 'cosmopolitan' MGEs that are widely distributed across host taxa contribute disproportionately to gene sharing, meaning that hosts carrying these genetic elements are likely to have a higher centrality within each multilayer mTPN. To account for this, and check that the relationships described above were not biased by urban conditions that favor cosmopolitan MGEs, each multilayer mTPN and GAM was reformatted using two reduced datasets; – *i)* a less stringent classification in which select MGEs that were found at a high frequency across taxonomic groups were removed, and *ii)* a more stringent and standardized classification, in which MGEs for which the highest proportion found in any one host taxa was less than 36% (when compared across other host taxa) were removed, and subsequently all MGEs with a variance of less than 0.01 between the relative proportion found in each of the four host taxa (wild birds, rodents, livestock and humans) were removed. Outputs for each of the three generalized additive models (Model 1 – complete MGE dataset unfiltered for cosmopolitan MGEs; Model 2 – singletons, doubletons and common MGEs removed; Model 3 – MGE's with a variance of less than 0.01 removed) are displayed in this table.

References

1. M. Wardeh, K. J. Sharkey, M. Baylis, Integration of shared-pathogen networks and machine learning reveals the key aspects of zoonoses and predicts mammalian reservoirs. *Proceedings of the Royal Society B: Biological Sciences* **287**, 20192882 (2020).
